# Multiplex Immunofluorescence Reveals Therapeutic Targets EGFR, EpCAM, Tissue Factor, and TROP2 in Triple-Negative Breast Cancer

**DOI:** 10.3390/ijms26157430

**Published:** 2025-08-01

**Authors:** T. M. Mohiuddin, Wenjie Sheng, Chaoyu Zhang, Marwah Al-Rawe, Svetlana Tchaikovski, Felix Zeppernick, Ivo Meinhold-Heerlein, Ahmad Fawzi Hussain

**Affiliations:** 1 Department of Gynecology and Obstetrics, Medical Faculty, Justus-Liebig-University Giessen, Klinikstr. 33, 35392 Giessen, Germany; tm.mohiuddin@mhb-fontane.de (T.M.M.); wenjie.sheng@med.uni-giessen.de (W.S.); chaoyuzhang81@gmail.com (C.Z.); marwah.al-rawe@gyn.med.uni-giessen.de (M.A.-R.); felix.zeppernick@gyn.med.uni-giessen.de (F.Z.); ivo.meinhold-heerlein@gyn.med.uni-giessen.de (I.M.-H.); 2 Department of Gynaecology and Obstetrics, Brandenburg Medical School Theodor Fontane, University Clinic Brandenburg, Hochstraße 29, 14770 Brandenburg an der Havel, Germany; svetlana.tchaikovski@mhb-fontane.de

**Keywords:** triple-negative breast cancer, EGFR, EpCAM, tissue factor, TROP2, multiplex immunofluorescence

## Abstract

Triple-negative breast cancer (TNBC) is a clinically and molecularly heterogeneous subtype defined by the absence of estrogen receptor (ER), progesterone receptor (PR), and human epidermal growth factor receptor 2 (HER2) expression. In this study, tumor specimens from 104 TNBC patients were analyzed to characterize molecular and clinicopathological features and to assess the expression and therapeutic potential of four key surface markers: epidermal growth factor receptor (EGFR), epithelial cell adhesion molecule (EpCAM), tissue factor (TF), and trophoblast cell surface antigen (TROP2). Multiplex immunofluorescence (mIF) demonstrated elevated EGFR and TROP2 expression in the majority of samples. Significant positive correlations were observed between EGFR and TF, as well as between TROP2 and both TF and EpCAM. Expression analyses revealed increased EGFR and TF levels with advancing tumor stage, whereas EpCAM expression declined in advanced-stage tumors. TROP2 and TF expression were significantly elevated in higher-grade tumors. Additionally, EGFR and EpCAM levels were significantly higher in patients with elevated Ki-67 indices. Binding specificity assays using single-chain variable fragment (scFv-SNAP) fusion proteins confirmed robust targeting efficacy, particularly for EGFR and TROP2. These findings underscore the therapeutic relevance of EGFR and TROP2 as potential biomarkers and targets in TNBC.

## 1. Introduction

Breast cancer remains the most commonly diagnosed cancer among women worldwide, with 2.3 million new cases and 670,000 deaths reported in 2022. By 2050, these figures are projected to increase by 38% and 68%, respectively [1]. Triple-negative breast cancer (TNBC) is a biologically aggressive subtype of breast cancer, characterized by the absence of estrogen receptor (ER), progesterone receptor (PR), and human epidermal growth factor receptor 2 (HER2) expression [2]. TNBC accounts for approximately 10–20% of all breast cancers cases [3] and is associated with a poor prognosis due to its high propensity for distant metastasis and limited treatment options, largely because of the absence of well-defined molecular targets [4]. Current therapeutic approaches rely primarily on chemotherapy, as TNBC typically exhibits limited responsiveness to endocrine or HER2-targeted therapy [5,6]. This presents a significant challenge in optimizing patient management.

In recent years, the advent of immunotherapy and Poly (ADP-ribose) polymerases (PARP) inhibitors has demonstrated modest benefits in select TNBC subgroups; however, considerable gaps remain in our understanding of actionable biomarkers and prognostic indicators for this heterogeneous disease [7,8,9]. Consequently, there is a growing interest in the molecular profiling of TNBC tumors to identify surface markers and signaling pathways that may serve as novel therapeutic targets.

Several surface molecules—including epidermal growth factor receptor (EGFR), epithelial cell adhesion molecule (EpCAM), tissue factor (TF), and trophoblast cell surface antigen (TROP2)—have attracted considerable attention for their potential roles in TNBC progression and therapeutic targeting [10,11,12,13]. EGFR is frequently overexpressed in breast cancer and associated with poor clinical outcomes, making it a potential target for therapeutic inhibition [14]. Although anti-EGFR therapies have shown limited efficacy overall, specific expression profiles may predict responsiveness to EGFR-targeted treatments; however, the prognostic significance of EGFR in TNBC remains unclear.

EpCAM contributes to cell adhesion, proliferation, tumor initiation, and epithelial–mesenchymal transition (EMT) [15]. While its expression in TNBC is variable, EpCAM overexpression is associated with poor prognosis and may indicate an aggressive tumor phenotype, suggesting its potential as a target for specific therapeutic approaches [13,16,17]. Aberrant TF expression is commonly observed in TNBC [18] and contributes to a hypercoagulable state that promotes fibrin formation, tumor cell survival, immune evasion, metastasis, and, ultimately, reduced patient survival [19,20,21,22]. Beyond its role in hemostasis, TF also promotes tumor growth, angiogenesis, and metastasis through signaling pathways such as prostate-activated receptor-2 (PAR2) and mitogen-activated protein kinase (MAPK) [20]. However, TF expression patterns in TNBC remain incompletely characterized. TROP2, a calcium signal transducer, is overexpressed in various epithelial cancers and contributes to tumor proliferation, invasion, migration, and survival through MAPK pathway activation [23]. Recently, TROP2 has emerged as a promising target for antibody–drug conjugate therapy in metastatic TNBC [24], and its expression may serve as both a prognostic and predictive biomarker [25]. Despite growing evidence for the individual importance of EGFR, EpCAM, TF, and TROP2 in TNBC biology, limited data are available on their co-expression patterns, mutual interaction, and relationships with clinicopathologic features such as HER2 and Ki-67 expression.

Tissue microarray (TMA) technology allows for high-throughput assessment of protein expression across large tumor cohorts using a minimal amount of tissue samples [26]. When combined with multiplex immunofluorescence (mIF), TMAs enable the simultaneous detection of multiple antigens within a single tissue core, preserving spatial context and tumor heterogeneity [27,28]. This approach is particularly well suited for investigating co-expression patterns, tumor microenvironment interactions, and the molecular diversity that characterizes TNBC.

In this study, we applied a multiplex TMA strategy to profile the expression landscape of EGFR, EpCAM, TF, and TROP2 in TNBC specimens and explored associations between their expression and clinicopathological features, including tumor grade, tumor stage, HER2 status, and Ki-67 expression.

## 2. Results

### 2.1. Patient and Tumor Characteristics

A total of 110 samples, 107 tumors and 3 non-tumor controls, were analyzed. Of the tumors, 104 samples were truly TNBC, 1 was triple-positive breast cancer, 1 was only Her2-positive, and 1 was Her2-positive + ER-positive + PR-negative. Based on pathological stage, 6 tumors were classified as stage I, 80 as stage II, and 21 as stage III (Table 1). Tumor grading revealed that 1 tumor was grade I, 42 were grade 2, and 64 were grade 3. HER2 expression levels were distributed as follows: 39 tumors were HER2-negative, 67 exhibited low HER2 expression, and 3 were HER2-positive. Regarding the Ki-67 proliferation index, 19 tumors were Ki-67 < 2%, 58 showed low Ki-67 expression (2–49%), and 33 demonstrated high Ki-67 levels (>50%).

### 2.2. Evaluating the Expression Pattern of EGFR, EpCAM, TF, and TROP2 in TNBC

**Figure 1:**
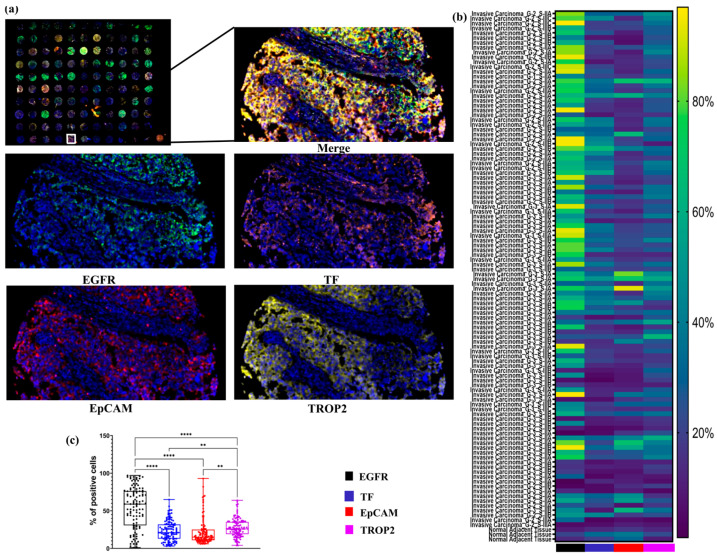
Expression profiles of EGFR, TF, EpCAM, and TROP2 in TNBC tissues. (**a**) Representative mIF images showing the expression of EGFR, TF, EpCAM, and TROP2 in TNBC issues (TMA BR1102). Images included full core scans of 110 samples and corresponding high-magnification (100×) views from selected regions, with each marker visualized in a distinct fluorescent channels. (**b**) Heatmap depicting the distribution and relative expression levels of EGFR, TF, EpCAM, and TROP2 across 110 different tissues cores, enabling comparative visualization of marker-positive cell proportion. (**c**) Boxplot illustrating the median, the interquartile range, and the individual percentage of cells positive for each marker across the 104 different TNBC tissues cores. **, *p* ≤ 0.01; ****, *p* ≤ 0.0001.

To assess the spatial and quantitative expression patterns of EGFR, EpCAM, TF, and TROP2 in TNBC tissues (BR1102, TissueArray.Com), a tyramide signal amplification (TSA)-based mIF protocol was employed. The staining revealed that tumors show frequent overexpression of all four investigated markers, with notable inter-sample variability (Figure 1).

A heatmap was used to visually display the proportion of marker-positive cells across all tissue samples, allowing for comparative analysis of expression patterns. Among the evaluated targets, EGFR and TROP2 emerged as the most abundantly expressed markers. EGFR, in particular, showed strong expression in invasive carcinoma samples, while TROP2 was expressed at moderate levels in the majority of samples.

Quantitative analysis confirmed that the proportions of EGFR- and TROP2-positive cells were significantly higher than those expressing EpCAM and TF across the entire TNBC cohort (Figure 1). These findings highlight EGFR and TROP2 as potentially valuable targets for diagnostic or therapeutic strategies in TNBC.

### 2.3. Correlation of the EGFR, EpCAM, TF, and TROP2 Expressionsw in TNBC

**Figure 2:**
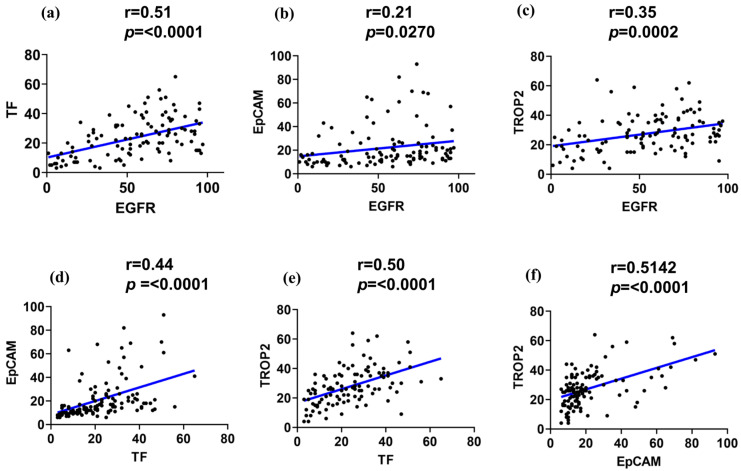
Correlation of the EGFR, TF, EpCAM, and TROP2 expressions in TNBC. Pearson correlation plots illustrate pairwise associations between the surface marker expression levels in TNBC tissue cores (*n* = 104). (**a**) EGFR vs. TF, (**b**) EGFR vs. EpCAM, (**c**) EGFR vs. TROP2, (**d**) TF vs. EpCAM, (**e**) TF vs. TROP2, and (**f**) EpCAM vs. TROP2. The blue line indicates the fitted linear regression line between the two markers.

To explore potential co-expression patterns among the four surface markers, we quantitatively analyzed their expression levels across 104 TNBC cores. Pearson correlation analysis revealed several statistically significant associations, suggesting coordinated expression of specific marker pairs (Figure 2a).

A strong positive correlation was observed between EGFR and TF expression (r > 0.51, *p* < 0.0001), suggesting potential biological interplay or co-regulation. Similarly, EGFR demonstrated a moderate positive correlation with TROP2 (r > 0.35, *p* < 0.0002). In contrast, EGFR and EpCAM showed only a weak correlation (r > 0.21, *p* < 0.0270). Notably, TF and EpCAM displayed a moderate correlation (r > 0.44, *p* < 0.0001), while TF and TROP2 exhibited a strong positive correlation (r > 0.51, *p* < 0.0001). Additionally, a significant positive correlation was also detected between EpCAM and TROP2 (>0.50, *p* < 0.0001), suggesting potential co-expression of epithelial and trophoblastic features in subsets of TNBC (Figure 2d–f).

### 2.4. Differential Expression Pattern of EGFR, EpCAM, TF, and TROP2 by Tumor Stage, Grade, and Patient Age in TNBC

**Figure 3:**
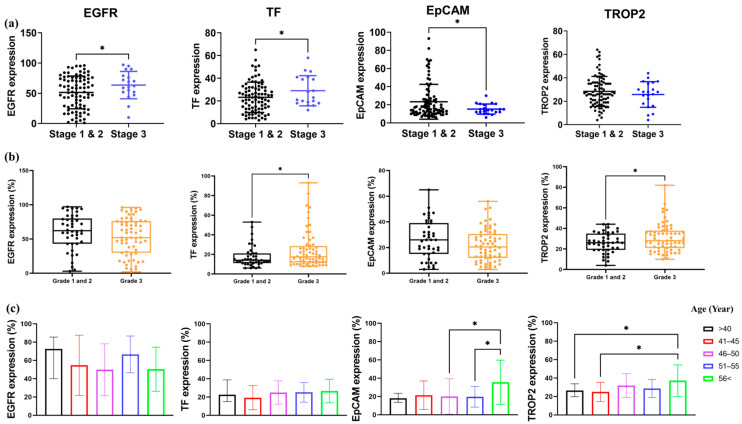
Differential expression of EGFR, TF, EpCAM, and TROP2 across TNBC stage, grade, and patient age. (**a**) Comparison of EGFR, TF, EpCAM, and TROP2 expression levels between early-stage (stage I–II) and advanced-stage (stage III) TNBC tissues. (**b**) Expression differences of the four markers between lower-grade tumors (grade 1−2) and high-grade tumors (grade 3). (**c**) Expression patterns of EGFR, TF, EpCAM, and TROP2 across five patient age groups. Boxplots display the distribution of marker-positive cell (%), and statistical significance was assessed using one-way ANOVA with post hoc Tukey’s test. Asterisks indicate levels of significance: *, *p* < 0.05.

To investigate the clinical relevance of EGFR, EpCAM, TF, and TROP2 expression, we stratified TNBC tissue samples by tumor stage (I–III), histological grade (G1–G3), and patient age and assessed marker distribution via mIF analysis. EGFR and TF expression levels were significantly elevated in stage III tumors compared to stage I and II samples (Figure 3a), suggesting a potential association with tumor progression and invasiveness. In contrast, EpCAM expression was markedly reduced in higher-stage tumors, indicating a possible loss of epithelial characteristics during tumor advancement. TROP2 expression, however, remained relatively consistent across all stages and did not show statistically significant variation.

In higher-grade tumors (G3), we observed a significant increase in TF and TROP2 expression in comparison to low-grade tumors (Figure 3b), suggesting their association with aggressive tumor phenotypes. In contrast, EGFR and EpCAM levels did not significantly differ across tumor grades.

Analysis of age-stratified groups revealed that EpCAM and TROP2 expression levels were significantly higher in patients aged ≥ 56 years compared to those ≤55 years (Figure 3c). No significant differences in EGFR and TF expression were observed between age groups.

These findings suggest that TF and TROP2 are associated with both higher tumor grade and stage, while EpCAM expression may decline with increasing tumor stage but increase with patient age. This highlights the complexity of marker regulation in TNBC and may facilitate future biomarker-based stratification strategies.

### 2.5. EGFR and EpCAM Expression Correlate with Proliferative Index but Not HER2 Status in TNBC

**Figure 4:**
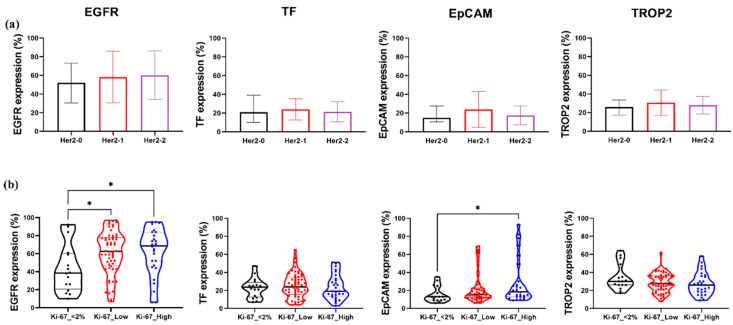
Expression patterns of EGFR, TF, EpCAM, and TROP2 in relation to HER2 and Ki-67 status in TNBC. (**a**) Comparison of EGFR, TF, EpCAM, and TROP2 expression levels across TNBC samples stratified by HER2 status: HER2-0, Her2-1+, and Her2-2+. (**b**) Expression analysis of the same four markers in TNBC samples grouped by Ki-67 proliferation index: Ki-67 < 2%, Ki-67 low (2–49%), and Ki-67 high (≥50%). Boxplots represent the distribution of marker-positive cells (%). Statistical significance was assessed using one-way ANOVA with Tukey’s post hoc test. *, *p* < 0.05.

To determine whether HER2 expression levels influence the expression of EGFR, TF, and EpCAM TROP2 in TNBC, patient samples were stratified into three subgroups: HER2-0, HER2-1+, and HER2-2+. No statistically significant differences were observed for any of the four markers across the HER2 expression groups (Figure 4a). Nonetheless, a trend toward elevated EGFR expression in HER2-2+ tumors compared to HER2-0 was noted, suggesting potential, though not significant, co-regulation in a subset of tumors.

We subsequently examined whether the expression levels of the four markers were associated with the Ki-67 index. Patients were grouped in Ki-67 < 2%, Ki-67 low (2–49%), and Ki-67 high (≥50%) categories, each containing ≥12 samples. EGFR expression was significantly increased in the Ki-67 high group compared to the Ki-67 < 2 tumors (Figure 4b), implicating EGFR in proliferative signaling in TNBC. Similarly, EpCAM levels were significantly elevated in Ki-67 high tumors, highlighting its potential association with increased cellular proliferation. In contrast, TF and TROP2 expression did not significantly differ across the Ki-67 groups.

### 2.6. Evaluating the Binding Specificity scFv-SNAP Fusion Proteins in TNBC Tissue

**Figure 5:**
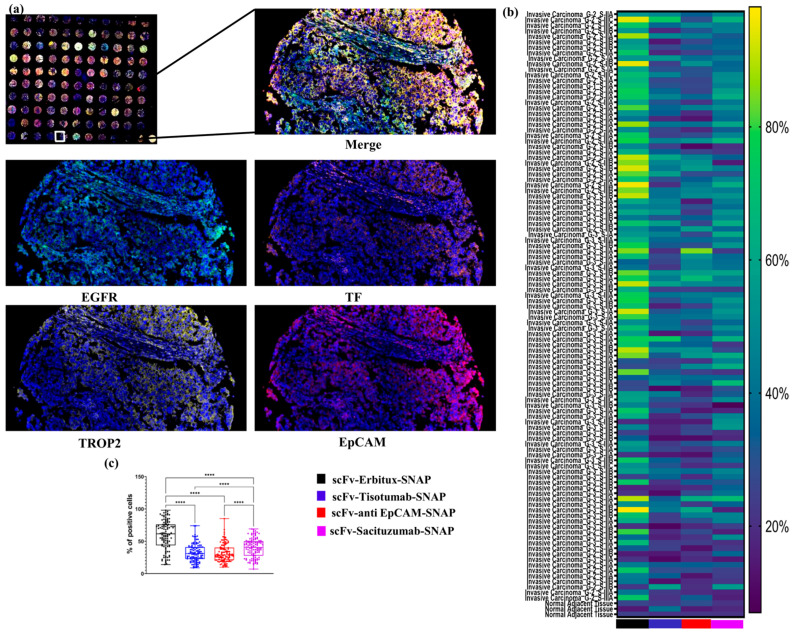
Binding specificity scFv-SNAP fusion proteins in TNBC tissue. (**a**) Microphotographs from mIF showing the binding specificities of four scFv-SNAP fusion proteins—scFv-Erbitux-SNAP, scFv-Tisotumab-SNAP, scFv-anti EpCAM-SNAP, and scFv-Sacituzumab-SNAP—across 110 tissue samples. Representative 100× images from a single core are shown for each fusion protein, with visualization across four fluorescence channels. (**b**) A heatmap depicting the percentage of cells bound by each scFv-SNAP fusion proteins in all 104TNBC tissues. (**c**) Boxplot illustrating the median, interquartile range, and individual percentage of cells binding for each scFv-SNAP fusion proteins across the tissue samples for each marker. ****, *p* ≤ 0.0001.

To assess the targeting efficacy in TNBC, we applied an mIF approach with scFv-SNAP fusion proteins. The resulting heatmap illustrates the binding intensity of each tissue core. Among the constructs tested, scFv-Erbitux-SNAP demonstrated the highest binding intensity across all TNBC cores, consistent with the EGFR expression profiles in these samples (Figure 5a). Additionally, scFv-Tisotumab-SNAP, scFv-anti EpCAM-SNAP, and scFv-Sacituzumab-SNAP exhibited moderate binding intensities, correlating with the moderate expression levels of TF, EpCAM, and TROP2, respectively. As shown in Figure 5c, the percentages of EGFR- and TROP2-positive cells were significantly higher than those of EpCAM- and TF-positive cells across all TNBC tissue samples. These findings underscore the effective and specific targeting capabilities of scFv-SNAP fusion proteins in TNBC tissues, particularly those directed against EGFR and TROP2.

## 3. Discussion

TNBC remains one of the most aggressive and clinically challenging subtypes of breast cancer, largely due to the absence of well-defined molecular targets [29,30]. In this study, we employed a TSA-based mIF approach to comprehensively assess the expression patterns of four surface antigens—EGFR, EpCAM, TF, and TROP2—in TNBC tissues. Our approach offers distinct advantages over conventional immunohistochemistry, particularly enabling simultaneous, high-resolution detection of multiple antigens within a single tissue microarray section [31]. This approach significantly enhances specificity through its use of HRP-mediated amplification and covalent tyramide deposition, minimizing background noise and cross-reactivity [32]. This results in improved signal intensity and detection sensitivity, making it especially efficient for identifying low-abundance targets [33]. Furthermore, the method exhibits robust reproducibility across samples and staining cycles, ensuring consistent data quality. Its compatibility with FFPE tissues and digital image analysis platforms further supports its promising clinical applicability [31,34], allowing for comprehensive spatial and quantitative assessment of biomarker expression patterns critical for precision oncology. Our results reveal distinct expression levels, co-expression relationships, and associations with clinicopathological parameters, providing insights that may guide the development of targeted therapeutic strategies. Consistent with previous studies, EGFR emerged as the most highly expressed marker in TNBC [12,35]. Across all patients, EGFR expression was more than twice as high as those of TF, EpCAM, and TROP2. This finding aligns with the well-established role of EGFR in TNBC pathogenesis, progression, and invasiveness [36]. TROP2 also demonstrated relatively strong expression, though at a lower extent than EGFR. A previous study demonstrated that 744 TNBC patients out of 807 TNBC patients showed moderate to strong TROP2 expression [37]. In contrast, TF and EpCAM were expressed at low, albeit detectable, levels in the majority of tumor regions. A previous study observed EpCAM expression in 660 out of 1365 cases (48%) and reported significant variation across the different intrinsic subtypes of breast cancer [38]. Another study reported that tissue factor (TF) was expressed in only 10% of breast cancer biopsies overall, but its frequency increased to 35% in the triple-negative breast cancer (TNBC) subgroup [39].

Beyond absolute expression, correlation analysis revealed significant co-expression patterns among the four surface markers. EGFR showed a strong positive correlation with TF and a moderate correlation with TROP2, indicating potential co-regulation or shared signaling pathways such as PI3K-AKT, MAPK/ERK, or EMT [40,41,42,43,44]. Similarly, Milsom et al. demonstrated that EGFR triggers the up-regulation of TF, which affects EMT modulation [41]. In contrast, EGFR’s correlation with EpCAM was weak, and this was also observed in another study [42]. Notably, TROP2 demonstrated moderate to strong correlations with EpCAM. Consistent with this, another study revealed frequent co-expression of TROP2 and EpCAM in cancers of the urinary bladder, prostate, pancreas, breasts, and ovaries [45]. These co-expression patterns may hold therapeutic relevance, as combinatorial targeting strategies could leverage these relationships to enhance treatment specificity and efficacy.

We further explored how expression levels of EGFR, EpCAM, TF, and TROP2 varied with clinical parameters such as tumor stage, grade, age, HER2 expression, and Ki-67 proliferation index. EGFR and TF expression were significantly elevated in stage III TNBC samples, suggesting their association with more advanced disease. This is consistent with reports showing dynamic TF expression in other cancers, such as non-small-cell lung cancer (NSCLC) [39]. Interestingly, EpCAM expression declined in advanced-stage disease, suggesting a potential role in earlier tumor development or loss of epithelial characteristics during tumor progression. However, other studies have reported increased EpCAM positivity in advanced-stage TNBC [46], highlighting the need for further investigation. TROP2 expression remained relatively stable across stages, suggesting consistent expression throughout TNBC evolution.

With respect to tumor grade, TF and TROP2 levels were significantly higher in poorly differentiated high-grade TNBC. These findings are consistent with studies showing elevated TF levels in advanced-stage and high-grade NSCLC [47,48]. In contrast, no significant grade-dependent differences were observed for EGFR or EpCAM in our cohort, although other studies have reported a positive association between EGFR expression and tumor grade [35]. These results suggest that EGFR expression may be more stage-dependent, while TF and TROP2 are more closely linked to histological differentiation and tumor aggressiveness.

When stratified by HER2 and Ki-67 status, we observed no significant differences in marker expression across the HER2-negative to low-expressing groups, although EGFR showed a trend toward higher expression in HER2-2 samples. This may suggest subtle crosstalk between the EGFR and HER2 pathway in a subset of TNBC patients, warranting further investigation. Notably, both EGFR and EpCAM expression were significantly elevated in patients with high Ki-67 indices, suggesting their potential roles in highly proliferative TNBC subtypes. In alignment with our findings, other studies reported the positive correlation of EpCAM expression with Ki-67 in gastric and hepatocellular carcinoma [49,50].

To evaluate the potential therapeutic relevance of investigated markers, we validated the binding specificity of scFv-SNAP fusion proteins using the TSA-based mIF method. These constructs showed selective binding to TNBC tissues, consistent with their corresponding antigen expression profiles. Compared to other constructs, scFv-Erbitux-SNAP exhibited 1.9-fold higher binding than scFv-Tisotumab-SNAP and scFv-anti-EpCAM-SNAP and 1.5-fold higher binding than scFv-Sacituzumab-SNAP. Similarly, our recent study demonstrated strong binding of scFv-Erbitux-SNAP and scFv-Sacituzumab-SNAP in breast cancer tissue [31]. Minor discrepancies between scFv and commercial antibody binding may result from differences in molecular structure, epitope accessibility, or the number of HRP molecules conjugated to each antibody. Nevertheless, these data support the high specificity and binding efficiency of the scFv-SNAP platform in TNBC tissues.

## 4. Materials and Methods

### 4.1. Tissue Microarray

The TMA used in this study (BR1102; TissueArray.Com, Derwood, MD, USA) comprised a total of 110 cores, including 104 samples from TNBC tissue, 1 triple-positive breast cancer sample, 1 HER2-positive + ER-positive + PR-negative sample, 1 HER2-positive sample, and 3 adjacent normal breast tissues. All TNBC specimens were obtained from female patients diagnosed with TNBC. Patient ages ranged from 27 to 86 years. Tumor grades ranged from 1 to 3, and tumor stages ranged from stage I to stage III. HER2 and Ki-67 expression status were provided by the supplier. A detailed breakdown of the TMA composition, including both normal and cancer tissues, is provided in the Results section.

### 4.2. Expression Analysis Using mIF

Expression profiling was performed on TNBC TMA (BR1102, TissueArray.Com) using the mIF protocol. TMA slides were initially incubated at 60 °C for 40 min to facilitate paraffin melting, followed by deparaffinization in xylene for 20 min. Tissue sections were rehydrated through a graded ethanol series—100% (twice for 5 min), 90% (5 min), 80% (5 min), and 70% (5 min)—and rinsed twice with deionized water.

Antigen retrieval was conducted by immersing the slides in sodium citrate buffer (pH 6.0) and heating them in a microwave for 20 min. To quench endogenous peroxidase activity, sections were incubated with 3% hydrogen peroxide for 10 min and subsequently washed three times with phosphate-buffered saline (PBS). Non-specific binding sites were blocked using a blocking buffer consisting of 10% goat serum in PBS for 1 **h** at room temperature.

Primary staining was performed by incubating the sections overnight at 4 °C in a humidified chamber with the polyclonal mouse anti-EGFR antibody (Sigma-Aldrich, Darmstadt, Germany, #HPA018530, 1:200 dilution). The following day, slides were equilibrated to room temperature for 1 h and washed three times with PBST (PBS containing 0.1% Tween 20). Subsequently, sections were incubated for 1 h with an HRP-conjugated secondary antibody (anti-mouse IgG, Invitrogen, #G21040), followed by three additional PBST washes. Signal amplification was achieved using Alexa Fluor 488 Tyramide (Invitrogen, B40953) for 10 min. After a final set of PBST washes, the slides were subjected to antigen stripping by microwave heating in sodium citrate buffer (pH 6.0) for 20 min to allow for subsequent rounds of staining.

Multiplex staining was then carried out sequentially using the following primary antibodies: anti-TF antibody (CD142 mAb, clone HTF-1, 10 µL, Miltenyi Biotec, Tokyo, Japan, #130-098-741), anti-EpCAM antibody (monoclonal antibody; mAb, 1:200, Invitrogen, Schwerte, Germany, #14-9326-82), and anti-TROP2 (mAb, clone MR54, 1:100, Invitrogen, #14-6024-82). Each primary antibody was subsequently subjected to incubation with an appropriate HRP-conjugated secondary antibody (anti-mouse or anti-rabbit, depending on host species). Tyramide signal amplification was performed using Alexa Fluor 546 Tyramide (Invitrogen, B40954), Alexa Fluor 647 Tyramide (Invitrogen, B40958), and iFluor 750 Styramide (AAT Bioquest, Pleasanton, CA, USA, #45065), respectively, following the same protocol as described for EGFR.

Finally, sections were mounted with a DAPI-containing antifade mounting medium (Vector Laboratories, Newark, CA, USA, #H-1200). Fluorescence imaging was conducted using the Axioscan 7 slide scanner (Zeiss, Oberkochen, Germany) with ZEN imaging software (ZEN 3.4). Quantitative image analysis was performed using the open-source digital pathology software QuPath (v0.5.1) [51].

### 4.3. Cell Culture Conditions

HEK293T cells were sourced from ATCC. HEK293T cells were cultured in RPMI 1640, both supplemented with 10% fetal bovine serum (FBS) and 100 U/mL penicillins/streptomycin (Thermo Fisher Scientific, Rockford, IL, USA). Zeocin (InvivoGen, Toulouse, France) (0.1 mg/mL) was used in HEK293T cultures during protein expression. All cells were incubated at 37 °C with 5% CO_2_.

### 4.4. Recombinant Protein Expression and Protein Enrichment

Four recombinant SNAP-tagged single-chain variable fragment (scFv-SNAP) proteins—scFv-Erbitux-SNAP, scFv-anti-EpCAM-SNAP, scFv-Tisotumab-SNAP and scFv-Sacituzumab-SNAP—were expressed in mammalian HEK293T cells with previously established protocols [52]. The coding sequences of the scFv and SNAP-tag domains were cloned into a modified pMS vector, derived from pSecTag2 [53]. To ensure efficient secretion, a murine immunoglobulin κ-chain leader sequence was placed upstream of the fusion constructs. Following transient transfection and incubation, culture supernatants containing secreted recombinant proteins were harvested and subjected to affinity purification using an Ni-NTA Superflow cartridge (Qiagen, Hilden, Germany) operated on an ÄKTA start chromatography system (GE Healthcare Bio-Sciences AB, Uppsala, Sweden). Protein purity and molecular weight were assessed by SDS-PAGE.

Expression and integrity of the SNAP-tagged fusion proteins were further validated by fluorescent-based labeling. Specifically, SNAP-tag functionality was confirmed via covalent labeling with SNAP-Surface^®^ Alexa Fluor^®^ 488 (New England Biolabs, Ipswich, MA, USA). The labeled proteins were visualized under UV illumination using a ChemiDoc XRS+ imaging system (BIORAD, Hercules, CA, USA), confirming successful expression and labeling efficiency.

### 4.5. Binding Analysis Using mIF

To evaluate the binding capacity of scFv-SNAP fusion proteins as part of the targeted drug delivery system, the constructs scFv-Erbitux-SNAP, scFv-anti-EpCAM-SNAP, scFv-Tisotumab-SNAP, and scFv-Sacituzumab-SNAP were conjugated to SNAP-biotin (New England Biolabs, Ipswich, MA, USA, #S9110S). The conjugation was carried out at a 1:3 molar ratio (protein:SNAP-biotin) by incubation at room temperature for 2 h. Unreacted SNAP-biotin was removed using 40K MWCO Zeba™ Spin Desalting Columns (Thermo Fisher Scientific, Waltham, MA, USA), according to the manufacturer’s instructions.

Subsequent tissue staining followed the previously described mIF protocol, with one modification: instead of a secondary antibody, streptavidin conjugated HRP was employed to detect biotin-labeled fusion proteins. Fluorescence imaging was performed using the Axioscan 7 slide scanner (Zeiss) and analyzed with ZEN software. Quantitative analysis of the fluorescence signal and spatial distribution of protein binding was conducted using QuPath [51].

### 4.6. Statistical Analysis

Statistical analyses were performed using GraphPad Prism 9.0.0 (GraphPad Software, San Diego, CA, USA). Heatmaps and expression comparisons were generated using one-way analysis of variance (ANOVA) followed by post hoc multivariate adjustment. Pearson correlation coefficients were calculated to assess expression correlations between markers. Comparisons between treatment groups and control groups were also conducted using one-way ANOVA followed by Tukey’s multiple comparisons test to determine statistical significance. Data are presented as mean ± standard error of the mean (SEM). A *p*-value < 0.05 was considered statistically significant.

## 5. Conclusions

In conclusion, this study underscores the complexity of surface antigen expression in TNBC and highlights the utility of high-resolution, multiplexed profiling to guide targeted therapy development. EGFR and TROP2 emerged as broadly expressed and therapeutically actionable targets, while TF and EpCAM showed potential in specific tumor contexts. Furthermore, our scFv-SNAP data confirm the specificity and effectiveness of this platform for in situ targeting, suggesting its potential for both diagnostic and therapeutic applications. These findings provide a foundation for patient stratification and the design of multi-targeted, personalized therapeutic strategies. Future studies involving larger patient cohorts, functional validation, and clinical outcome data will be essential for translating these insights into effective clinical impacts.

## Figures and Tables

**Table 1:** Characteristics of study population.

Characteristics		Values
Sex	Female	110
Age	Median	48
	Range	27–86
Stages	Stage I	6
	Stage II	80
	Stage III	21
Grades	Grade 1	1
	Grade 2	42
	Grade 3	64
Malignant invasive carcinoma		107
Her2 status	Her2-0	39
	Her2-1	46
	Her2-2	21
	Her2-3	3
Ki-67 status	<2%	19
	≤49%	58
	≥50%	33
ER status	negative	105
	positive	4
PR status	negative	106
	positive	3

## Data Availability

The original contributions presented in this study are included in the article. Further inquiries can be directed to the corresponding author.
